# Mammal-infecting DNA viruses identified in lemurs and rodents in Madagascar mirror the evolutionary history of their hosts

**DOI:** 10.1099/mgen.0.001728

**Published:** 2026-05-22

**Authors:** Elise N. Paietta, Rachel A. Johnston, Simona Kraberger, Santatriniaina F. Randrianarisoa, Tahina T. Razanamahenina, Antsa Ramboninarimalala, Jean-Baptiste Velontsara, Toussaint G. Raherinirina, Laurent Raveloson, Nina L. Finley, Eric Baitchman, Brian G. McAdoo, Anne D. Yoder, Arvind Varsani

**Affiliations:** 1 The Biodesign Center for Fundamental and Applied Microbiomics, Center for Evolution and Medicine, School of Life Sciences, Arizona State University, Tempe, AZ 85287, USA; 2 Department of Biology, Duke University, Durham, NC 27708, USA; 3 Zoo New England, Boston, MA 02121, USA; 4 Broad Institute of MIT and Harvard, Cambridge, MA 02142, USA; 5 Mahaliana Labs SARL, Amboditsiry, Antananarivo 101, Madagascar; 6 Department of Zoology and Animal Biology, University of Antananarivo, Antananarivo, Madagascar; 7 Centre ValBio, Ranomafana, Madagascar; 8 Health In Harmony-Fahasalamana Mirindra, Farafangana, Madagascar; 9 Department of Disease Control, London School of Hygiene and Tropical Medicine, London, WC1E 7HT, UK; 10 Earth and Climate Sciences Division, Nicholas School of the Environment, Duke University, Durham, NC 27708, USA; 11 Structural Biology Research Unit, Department of Integrative Biomedical Sciences, University of Cape Town, Cape Town 7925, South Africa

**Keywords:** *Avahi *sp., DNA viruses, *Eliurus minor*, *Eliurus webbi*, lemur, Madagascar, *Microcebus *sp., *Rattus rattus*, rodent, *Varecia variegata*, viral metagenomics

## Abstract

Given that some DNA viruses have been found to exhibit virus–host co-evolution and establish lifelong infection, mammals with unique evolutionary histories in island ecosystems likely host exceptionally diverse viruses. Madagascar is inhabited by endemic non-human primate and rodent lineages interacting with expansive populations of introduced non-native rodents across the island. Using a viral metagenomic workflow on 189 oral swabs of lemurs and rodents in southeastern Madagascar, we characterized genomic sequences of DNA viruses in the families *Adenoviridae*, *Circoviridae*, *Orthoherpesviridae*, *Papillomaviridae*, *Parvoviridae* and *Polyomaviridae* and assessed their phylogenetic relationships to known viruses. Endemic lemurs and tufted-tailed rats displayed particularly novel DNA viral diversity mirroring the geographic isolation and subsequently rich evolutionary history of their hosts. Notably, we provide the first coding-complete sequences in lemurs of herpesviruses, polyomaviruses, adeno-associated viruses and circoviruses. In contrast, the DNA viral communities of black rats in Madagascar were similar to those found in globally distributed black and brown rat populations, given their broad geographic spread and relatively recent introduction to the island. Given the scarcity of viral research in natural populations of lemurs and rodents in Madagascar despite the island’s exceptional biodiversity and escalating anthropogenic pressures, this study provides a genomic and phylogenetic foundation for DNA viruses infecting Malagasy lemurs and rodents.

**Impact Statement**

Despite the incredible endemicity, evolutionary history and population decline of most mammals on the island of Madagascar, associated viral genomic resources for wildlife across this globally significant biodiversity hotspot have remained scarce. In this study, metagenomic analysis focusing on DNA of oral swabs across an array of mammalian species in southeastern Madagascar provided a unique opportunity to examine how the rich evolutionary history of endemic lemurs and tufted-tailed rats, compared with the more recent introduction of widespread non-native black rats, impacts the diversity of mammal-infecting DNA viruses and demonstrates patterns of virus–host co-evolution. We identified viral sequences spanning six families (*Adenoviridae*, *Circoviridae*, *Orthoherpesviridae*, *Papillomaviridae*, *Parvoviridae *and *Polyomaviridae*), including those representing two new circovirus species, three new herpesvirus species, two new papillomavirus species, three new parvovirus species and one new polyomavirus species, some of which additionally represent putative new genera. By integrating genomic characterization with detection frequency and co-infection data, this study substantially expands DNA virus resources for Madagascar’s mammals and provides new insights into how long-term geographic isolation and non-native rodent introductions shape viral diversity in mammals.

## Data availability

The virus sequences described in this study have been deposited in GenBank under accession numbers PX705241–PX705265 and PZ014654. Raw read sequence data for samples have been deposited under BioProject number PRJNA1290322, BioSample numbers SAMN49916477–SAMN49916666 and SRA numbers SRR34496596–SRR34496785.

## Introduction

Biodiversity hotspots, defined as ‘exceptional concentrations of endemic species undergoing exceptional loss of habitat’, represent areas with frequent contact between endemic and non-native animal populations and human communities [1]. Some of these biodiversity hotspots are islands (e.g. Madagascar, Polynesia/Micronesia, the Caribbean, the Philippines, New Caledonia and New Zealand), which, prior to widespread human influence, presented long-term environmental stability, geographical isolation and unique ecological attributes, providing the time and space for the evolution of large endemic mammalian lineages (e.g. the lemuriform primates and Nesomyinae rodents of Madagascar) [1,2]. Documentation of viral diversity in non-human primates and rodents in island biodiversity hotspots will enhance critical understanding of how viral evolution has been shaped alongside unique mammalian evolution. Further, investigating viral diversity in non-native mammals that have persisted and expanded on previously isolated biodiversity hotspot islands may help us to understand how both host taxonomy and exposure to unique environments may influence viral communities in widespread, non-native host species and the naïve, endemic host communities with which they interact.

Although more than 90% of publicly available viral sequences are from humans and four domestic ungulate and avian genera (*Anas*, *Bos*, *Gallus* and *Sus*) [3], recent studies targeting diverse animals for high-throughput sequencing and metagenomic discovery have led to breakthroughs in our understanding of evolutionary processes in diverse RNA and DNA viral families [4]. Some DNA viruses frequently demonstrate high host specificity, co-evolving with their hosts through evolutionary time while exacting lifelong infections. Long-term infections, including those with pathogenic or non-pathogenic outcomes, are an important factor increasing the chance of detection through metagenomic methods. Further, while much viral metagenomic research has focused on faecal samples, samples from the oral cavity have proved particularly useful for uncovering novel mammal-infecting DNA viral diversity from non-human primates and rodents [5,10]. Oral swab samples are rising as a diagnostic and discovery tool, providing insight into viruses replicating in epithelial tissues or being shed through saliva [11]. Here, we focus on DNA viruses found in oral swabs of mammals in Madagascar, a biodiversity hotspot with ~90% endemicity of animals and plants. Madagascar’s extraordinary diversity and long isolated evolutionary history have likely resulted in exceptionally diverse viruses circulating in the animals.

Over ~65 million years across Madagascar [12], lemurs evolved into one of the most speciose non-human primate lineages, composed of over 100 extant lemur species divided into 5 families (Cheirogaleidae, Daubentoniidae, Indriidae, Lemuridae and Lepilemuridae). As ~98% of lemur species are currently threatened with extinction [13], viruses may pose a significant threat to lemur conservation. Yet, viral research in non-human primates has been largely biassed towards Old World monkeys and great apes, neglecting primate lineages including lemurs, gibbons, siamangs, galagos, lorises and tarsiers. Research on DNA viruses in the lemuriform primates has been remarkably limited with the exception of previous studies on adenoviruses and anelloviruses in natural lemur populations [14,18] and diverse viral communities in captive lemurs, which serve as a comparison for work in Madagascar [7,8, 19,21]. Lemur adenoviruses and anelloviruses were divergent, forming lemur-specific lineages [14,18, 22].

Rodents are incredibly diverse (>2,000 species) and abundant, having global distributions and adapting readily to an array of environments from rainforests to urban centres [23]. Research on viruses in rodents has primarily focused on their role in harbouring zoonotic viral pathogens such as those causing haemorrhagic fevers in humans (e.g. hantaviruses and arenaviruses) [24], with some studies reporting that ~60% of zoonotic viruses originate in rodents [25]. Rodents can serve as bridges between anthropogenic and natural landscapes, with non-native rodents thriving across both settings. Non-native rodents, in particular black rats (*Rattus rattus*), brown rats (*Rattus norvegicus*) and house mice (*Mus musculus*), alter ecosystem dynamics across the planet by outcompeting endemic small mammals for resources and frequently moving between natural and human-inhabited spaces, especially in island settings [26]. Although black rats have been identified as one of the top mammals for harbouring zoonoses, they display significantly fewer viral sequence records than house mice and brown rats [25]. Black rats are estimated to have been introduced to Madagascar ~1,000 years ago through the Arabian trade network with multiple potential invasion scenarios [27,28]. Rodent virome studies have revealed distinct temporal and seasonal changes in viral richness and composition [29,30] and the role of rodent taxonomy over geographic location [31]. Madagascar presents complex interactions between non-native rodents, including black rats, brown rats and house mice, and Madagascar-specific rodent lineages of the subfamily Nesomyinae such as the tufted-tailed rats of the *Eliurus* genus. Tufted-tailed rats have evolved on Madagascar over ~13–15 million years with the *Eliurus* genus currently composed of 13 described species [32,33]. The few viral sequences in the public database from *Eliurus* spp. include hantaviruses and paramyxoviruses, viral families with known zoonotic members, highlighting interest in studying tufted-tailed rats.

In this study, we document expansive DNA viral diversity in oral swab samples of natural and frequently interacting populations of woolly lemurs, mouse lemurs, black-and-white ruffed lemurs, tufted-tailed rats and black rats in and around the Manombo Special Reserve (MSR) in southeastern Madagascar. Here, we focus on characterizing viruses in mammal-infecting DNA viral families that have previously been found to demonstrate virus–host co-evolution and induce long-term infections in their hosts. Further, our work evaluates how the rich evolutionary history of endemic lemurs and tufted-tailed rats, compared with the more recent introduction of widespread non-native black rats, impacts the diversity of mammal-infecting DNA viruses in southeastern Madagascar. Overall, given the evolutionary coupling between many DNA viruses and their hosts, we expect the biodiverse and previously isolated landscapes of Madagascar to be a hotspot for viral diversity.

## Methods

### Sample collection

The MSR, situated along the coast of southeastern Madagascar, south of the city of Farafangana, encompasses lowland rainforest and littoral forest parcels and is surrounded by ~31 villages with varying distances from the forest. Oral swabs (in addition to other sample types that are not part of this study) were collected from animals in the MSR and surrounding area in Madagascar during two expeditions, in October 2022 and July 2023, according to methods approved by the Duke University Institutional Animal Care and Use Committee under Protocol Registry Number A075‐23‐03 and the Zoo New England Institutional Animal Care and Use Committee under Protocol Number 2022-56. In addition, the described procedures were approved by the Madagascar government under permits n^o^ 286/22/MEDD/SG/DGGE/DAPRNE/SCBE.Re and n^o^ 215/23/MEDD/SG/DGGE/DAPRNE/SCBE.Re. The animals were sampled as described in Paietta *et al*. [18,34].

Oral swabs were collected with sterile flocked swabs (Puritan Medical Products, USA) and stored in 1 ml of Universal Transport Medium (Puritan UniTranz-RT, Puritan Medical Products, USA). The oral swab sample set analysed for this study was comprised of 45 lemur samples (8 *Avahi* sp. (woolly lemur)*,* 27 *Microcebus* sp. (mouse lemur), 10 *Varecia variegata* (black-and-white ruffed lemur)), 125 non-native *Rattus rattus* (black rat) samples, 9 *Eliurus webbi* (Webb’s tufted-tailed rat) samples and 5 *Eliurus minor* (lesser tufted-tailed rat) samples. Lemurs and endemic and non-native rodents were sampled in the lowland rainforest (6 sampling days per expedition) and littoral forest (6 sampling days per expedition) of the MSR. Non-native rodents were additionally sampled in villages (in and under homes, in cropping areas) (2 sampling days per expedition). All endemic animals were released at the GPS (Global Positioning System) point of the capture site after biological sampling. Non-native black rats were euthanized per American Veterinary Medical Association guidelines and Madagascar National Parks recommendation. Oral swab samples were kept on ice until being flash-frozen in liquid nitrogen at the end of the sampling day. Samples were maintained in liquid nitrogen during transport to Duke University (Durham, NC, USA) and stored in a −80 °C freezer until downstream processing.

### Nucleic acid extraction, library preparation and sequencing analyses

DNA was extracted from 200 µl of each sample with the Roche HighPure Viral Nucleic Acid Kit (Roche Diagnostics, USA) to enhance viral nucleic acid representation. DNA extracts were amplified using the Templiphi (Cytiva Life Sciences, USA) rolling circle amplification kit to target circular DNA viruses. A combination of rolling circle amplified and non-amplified DNA extract (50:50) was used as input to generate Illumina sequencing libraries (2×150 bp) using the Illumina DNA Prep (M) Tagmentation Kit (Illumina, USA). These libraries were sequenced in multiplex mode on the NovaSeq X Plus with Psomagen Inc. (USA). Paired-end reads were trimmed using Trimmomatic-0.39 [35] and *de novo* assembled with MEGAHIT v1.2.9 [36]. Circular contigs were identified based on terminal redundancy, and all contigs >1,000 nt were analysed for viral-like sequences using Diamond [37] blastx against a local viral RefSeq database. Viral genomes were annotated using CenoteTaker3 [38,39] and subsequent manual curation. CheckV [40] was used to further assess sequence completeness and quality using both an average amino acid identity (AAI)-based approach which output an expected genome length, completeness estimate, alignment fraction, confidence level and relative error in addition to a hidden Markov model (HMM)-based approach which reports a lower and upper bound of completeness. For the assembled virus genomes, we used BWA [41] to map the raw reads to the genomes with an aim to determine coverage and identify issues, including abrupt drops in coverage or bimodal coverage along a single contig, that could result from misassembly. For circular sequences with lower coverage and read count, we designed abutting primers, amplified the circular molecule, cloned the amplicon into the pJET1.2 plasmid (Thermo Fisher, USA) and sequenced with Plasmidsaurus (USA). In each case, the sequences obtained matched the original contig. PX705251 and PX705255 (Avahi polyomavirus 1) were amplified with abutting primer pair, F: 5′- AAATCTCTCAGATAACAGATCCTCCC-3′ and R: 5′- ACAATTATGTTGGAAAGACTTGGGG-3′. PX705254 (Avahi papillomavirus 1) was amplified with abutting primer pair, F: 5′- CCATGGGGCAATTATAAATTTTGGG-3′ and R: 5′- ATCTTTCTTTTCTGCAGGAACAACC-3′.

### Phylogenetic analyses

Datasets of protein sequences from representative viruses in established virus species in each viral family (taken from VMR_MSL39.v4_20241106) were assembled. These together with protein sequences of viruses identified in this study were used for downstream sequence analysis. Homologous proteins were aligned using MAFFT [42]. Maximum likelihood phylogenetic trees were constructed using IQ-TREE2 [43], employing the ModelFinder [44] option to select the best-fit amino acid substitution model and assessing branch support with the approximate likelihood ratio test (aLRT [45]; 10,000 replicates) and ultrafast bootstrap approximation (UFBoot [46]; 10,000 replicates). Values for aLRT (above) and UFBoot (below) on branches are both shown on each tree. For partitioned maximum likelihood phylogenetic trees (*Papillomaviridae*, *Orthoherpesviridae*), separate datasets and alignments were generated for each protein to be included and subsequently concatenated. All phylogenetic trees were rooted with representative outgroup sequences.

DNA polymerase amino acid sequences and best-fit model LG+F+R6 were used for the adenoviruses. The major capsid protein, uracil DNA glycosylase and ribonucleoside-diphosphate reductase amino acid sequences and best-fit models LG+F+R6, LG+F+R5, LG+F+R5 and LG+I+G4, respectively, were used for herpesviruses. Concatenated E1, E2 and L1 amino acid sequences and best-fit models LG+F+R8, LG+F+R9 and LG+F+R7, respectively, were used for the papillomaviruses (PVs). Large tumour antigen (LTag) amino acid sequences and best-fit model LG+F+R5 were used for polyomaviruses (PyVs). NS1 amino acid sequences and best-fit model LG+F+R6 were used for the parvoviruses. Replication-associated protein (Rep) amino acid sequences and best-fit model LG+R6 were used for circoviruses. Pairwise identity calculations between known and novel viruses were determined with the Sequence Demarcation Tool v1.241 [47] to determine sequence similarity. Genome organization and protein sequence comparison of viruses identified in this study and their most closely related sequences were generated using Clinker [48]. The International Committee on Taxonomy of Viruses (ICTV) VMR species [49] lists and criteria were used to determine established and novel species and genera.

### Distribution of virus genomes across samples

CD-Hit [50] was used to cluster virus genomes with greater than 95% average nucleotide identity into distinct virus operational taxonomic units (vOTUs). Using CoverM [51], Illumina sequencing raw reads were then mapped to a representative genome from each identified vOTU to determine the presence/absence of these vOTUs across all samples with ≥75% genome coverage as a high-confidence proxy of vOTU presence in a sample. Presence/absence data was then used to determine the detection frequency of each vOTU identified in this study and to identify co-infections.

## Results and discussion

### Identification of viruses in lemur and rodent oral swab samples

**Fig. 1.**
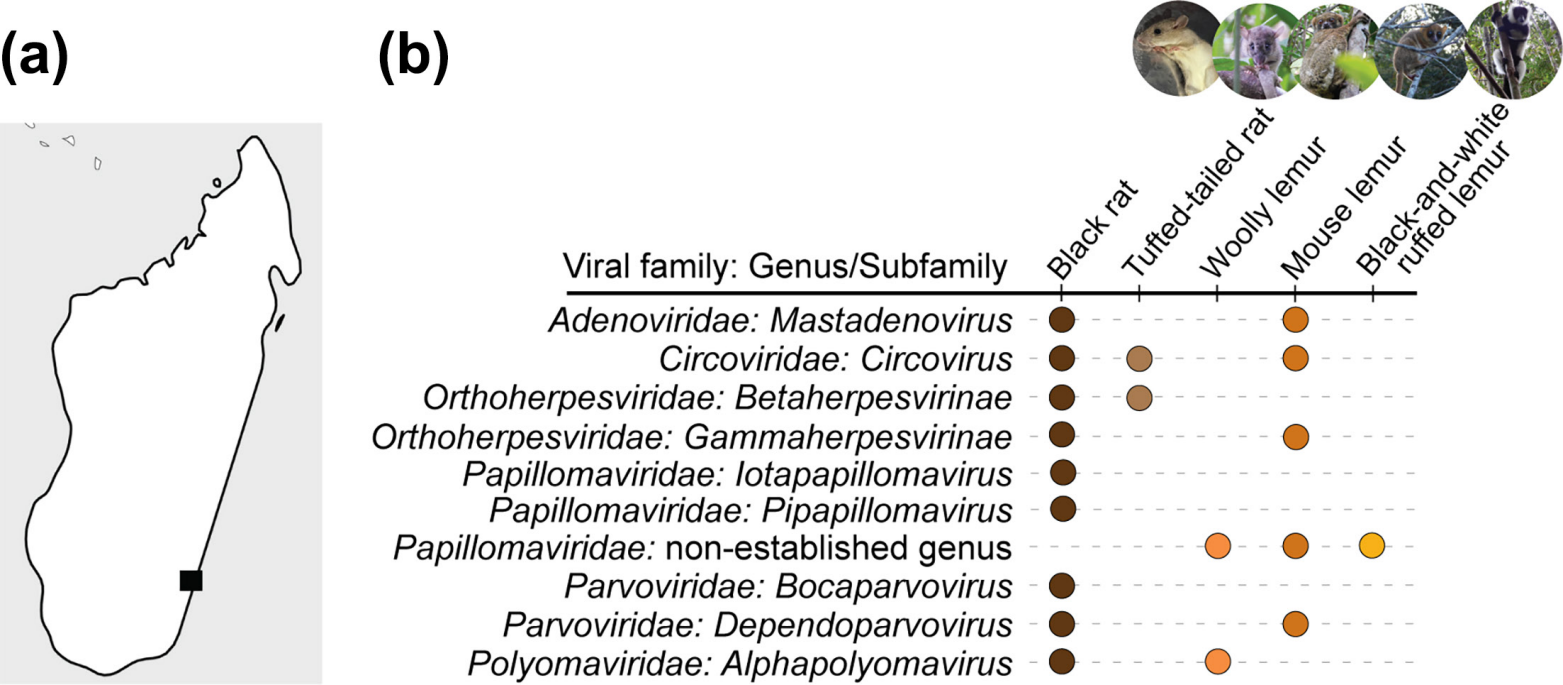
(**a**) Map of Madagascar with a black square denoting the location of the MSR in southeastern Madagascar. (**b**) Summary of mammal-infecting DNA virus taxa identified in this study from endemic lemurs, tufted-tailed rats and non-native black rats in the MSR.

The analysis of the sequencing results from the DNA extracts of 189 oral swabs from mouse lemurs (*n*=28), black-and-white ruffed lemurs (*n*=10), woolly lemurs (*n*=8), lesser tufted-tailed rats (*n*=5), Webb’s tufted-tailed rats (*n*=9) and black rats (*n*=129) at the MSR resulted in the identification of high-quality viral genome sequences that are part of the *Adenoviridae*, *Circoviridae*, *Orthoherpesviridae*, *Papillomaviridae*, *Parvoviridae* and *Polyomaviridae* families (Figs 1a, b; Fig S1, Supplementary Material 1; Sheets S1 and S2, Supplementary Material 2). Adenovirus, herpesvirus and parvovirus sequences were identified from black rats and mouse lemurs. PV sequences were identified from woolly lemurs, mouse lemurs, black-and-white ruffed lemurs and black rats. PyV sequences were characterized from woolly lemur and black rat samples. Circovirus sequences were identified from tufted-tailed rat, black rat and mouse lemur samples. Animals with viruses identified in oral swabs appeared healthy during the physical examinations conducted by a veterinarian at the time of sampling, with normal temperature, heart and respiratory rates, body and fur scores and non-diarrhoeal stool.

Mean coverage and read count for adenoviruses and herpesviruses ranged from 7.5 to 367.0 and 1,681 to 284,502 (Supplementary Material 1). Mean coverage and read count for PVs and PyVs ranged from 3.5 to 6,527.7 and 119 to 287,240 (Supplementary Material 1). Mean coverage and read count for parvoviruses ranged from 135.1 to 282.8 and 4,219 to 8,106 (Supplementary Material 1). Mean coverage and read count for circoviruses ranged from 15.1 to 368,426.1 and 195 to 3,745,156 (Supplementary Material 1). A few sequences had lower read counts including Avahi polyomavirus 1 (PX705251, PX705255) and Avahi papillomavirus 1 (PX705254) and were thus PCR-amplified to confirm the sequence. The amplicon matched the original contig for each. Twenty-three sequences were determined to be of high confidence for completeness via the AAI-based approach (Sheet S2, Supplementary Material 2). Three sequences were determined to be of medium confidence, although these represent three of the most divergent sequences in our dataset and are confirmed to be of high quality through the HMM-based approach (Sheet S2, Supplementary Material 2). No flags of misassembly or bimodal coverage were seen. No gaps were present in viruses with smaller genome sizes less than 10 kb in length (i.e. PVs, PyVs, parvoviruses and circoviruses). However, the parvovirus sequences are likely missing the ends of the genomes as these are formed by palindromic sequences able to be folded into hairpin structures and are difficult to *de novo* assemble. The circular genomes of PVs, PyVs and circoviruses were confirmed through terminal redundancy. For adenoviruses (~35 kb in length) and herpesviruses (~120–240 kb in length), these sequences can be more difficult to assemble given their larger genome sizes. Three sequences (one adenovirus, two herpesviruses) contained short gaps where larger contigs were joined with a series of Ns (red regions, Supplementary Material 1). Contigs were only joined if we did not find any other variant of the virus taxon group in that sample and have high confidence in joining. Black rat adenovirus 1 (PX705247, 34,458 nt in length) has gaps in nucleotide positions 24,961 to 25,030 and 26,921 to 27,132. Black rat herpesvirus 1 (PX705242; 212,811 nt in length) has gaps in nucleotide positions 56,623 to 56,675; 67,850 to 67,891; and 174,916 to 174,933. Black rat herpesvirus 2 (PZ014654; 131,431 nt in length) has a gap in nucleotide position 15,585 to 15,604. Additionally, tufted-tailed rat herpesvirus 1 (PX705242) is missing the terminal repeat region.

### Adenoviridae

Viruses classified in the family *Adenoviridae* have non-enveloped virions that encapsidate linear, dsDNA genomes ranging from 24 to 48 kb in length [52]. Although many adenovirus infections are subclinical, adenoviruses are known to affect their host’s respiratory systems. In some cases, they can induce severe or fatal upper respiratory tract infections [53]. Adenoviruses can be transmitted through a variety of routes including aerosolized droplet, faecal–oral and fomite pathways [54]. The *Adenoviridae* family is composed of six genera: *Mastadenovirus*, *Aviadenovirus*, *Ichtadenovirus*, *Testadenovirus*, *Siadenovirus* and *Barthadenovirus* [52]. The exclusively mammal-infecting viruses in the genus *Mastadenovirus* are thought to be specific to closely related species [52] and to exhibit virus–host co-evolution [14,55], although cross-species transmission is possible [56,58].

**Fig. 2.**
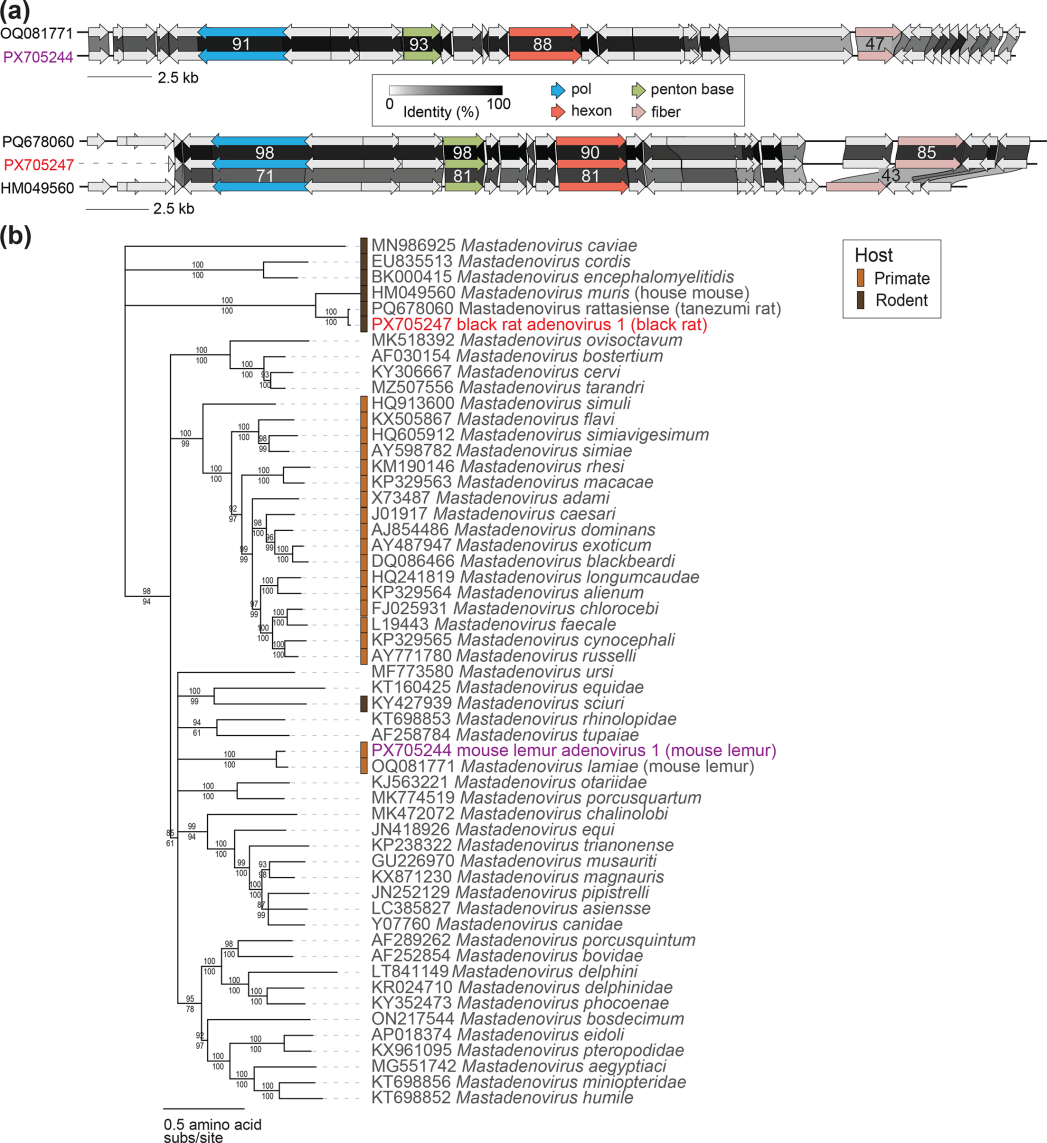
(**a**) Genome organization and encoded protein sequence comparison of adenoviruses identified in this study and their most closely related sequences. Percent amino acid pairwise identity values are shown for select proteins (DNA polymerase, penton, hexon and fibre proteins). (**b**) Maximum likelihood phylogenetic tree of DNA polymerase amino acid sequences with representatives of established species in the *Mastadenovirus* genus in addition to adenovirus sequences identified in this study. The phylogenetic tree is rooted with aviadenovirus DNA polymerase sequences. Red or purple font colours signify viral sequences identified in this study from rodents (red) and lemurs (purple). Percent aLRT branch (above) and UFBoot (below) support are shown for each branch.

Genome sequences of mastadenoviruses (lemur mastadenovirus, OQ081771–OQ081775) have previously been identified from mouse lemurs (*Microcebus griseorufus* and *Microcebus murinus* faeces) [14]. Additionally, mastadenovirus IVa2 gene fragments (~250 nt) are available in the public database for multiple lemur species in the Lemuridae family [55]. Here, we identified one coding-complete mouse lemur adenovirus sequence, which we name mouse lemur adenovirus 1 (PX705244; 36,366 nt). Mouse lemur adenovirus 1 shares similar genome organization and ~77% genome-wide pairwise identity with previously published mouse lemur adenovirus genomes (OQ081771–OQ081775) [14] (Fig. 2a). We then compared the IVa2 gene sequence of mouse lemur adenovirus 1 with IVa2 sequences available for multiple lemur genera; mouse lemur adenovirus 1 IVa2 gene sequences share 91.2% nucleotide identity with complete IVa2 sequences from lemur mastadenovirus isolate 2019 (OQ081775) from *M. griseorufus* [14] and 76.7–80.2% nucleotide identity with the IVa2 gene fragments from various Lemuridae species [55]. Adenovirus species are determined by a 15% DNA polymerase amino acid identity threshold, among other factors such as host range and genome organization [52]. Mouse lemur adenovirus 1 DNA polymerase shares 90.5–90.8% amino acid identity with the DNA polymerase amino acid sequences of mouse lemur adenoviruses (OQ081771–OQ081775) and clusters phylogenetically with members of the *Mastadenovirus lamiae* species [14] (Fig. 2b), supporting its classification within this established species. As mouse lemur adenovirus 1 shares greater similarity with adenoviruses from Cheirogaleidae species than from Lemuridae species, this likely suggests virus–host co-evolution among lemur adenovirus lineages with their host taxa.

Importantly, all established primate-infecting (human, chimp, macaque, baboon, African green monkey, golden snub-nosed monkey and titi monkey) mastadenovirus species cluster together as one large clade with the exception of the lemur mastadenoviruses, suggesting that in addition to adenovirus-lemur co-evolution, a historical host switch from a non-lemur host to a lemur host is possible (Fig. 2b) [14]. Lemur mastadenoviruses’ distant phylogenetic position relative to other primate mastadenovirus lineages reflects lemurs’ unique evolutionary history and geographic isolation in Madagascar [14].

Mastadenoviruses of various *Rattus* species including lesser rice field rats, brown rats, black rats and Tanezumi rats have been previously determined [59,62]. Here, we present a partial black rat adenovirus genome sequence named black rat adenovirus 1 (PX705247; 34,458 nt). Black rat adenovirus 1 shares 89.9% genome-wide similarity and 98% DNA polymerase amino acid identity (Fig. 2a) with the sequence of Yunnan rodent mastadenovirus 1 (PQ678060) from a Tanezumi rat gut sample from China [59] (Fig. 2a). Black rat adenovirus 1 shares 71% DNA polymerase amino acid identity with the representative of the closest established species *Mastadenovirus muris* (murine adenovirus 2 isolate K87, HM049560 [61]), and the sequence phylogenetically clusters with a rodent-specific mastadenovirus clade (Fig. 2a, b). Based on these results, black rat adenovirus 1 will be designated as a member of a proposed new species *Mastadenovirus rattasiense*, which includes Yunnan rodent mastadenovirus 1 (PQ678060) [59]. The high similarity between black rat adenovirus 1 and rat adenovirus sequences from *Rattus* species throughout Asia emphasizes the highly similar adenovirus communities in geographically widespread non-native rodents.

### Orthoherpesviridae

Viruses in the *Orthoherpesviridae* family infect reptiles, avians and mammals, having co-evolved alongside their hosts while successfully establishing lifelong latent infections [63]. Although infections are typically asymptomatic, these viruses can be oncogenic and capable of causing an array of clinical manifestations of disease (e.g. blisters, fever, swollen lymph nodes and cancer), in humans particularly impacting infants and immunocompromised individuals. Viruses in the *Orthoherpesviridae* family have large linear, dsDNA genomes ~120–240 kb in length [63]. Orthoherpesviruses are divided into three subfamilies, *Alphaherpesvirinae*, *Gammaherpesvirinae* and *Betaherpesvirinae*, having distinct host ranges, genomic architecture, replication and pathogenesis [63,65].

In the context of lemurs, a study identified herpesvirus DNA polymerase sequences (472–480 bp) for cheirogaleid herpesvirus 1 (KT698104, KT698105) and cheirogaleid herpesvirus 2 (KT698106) in liver samples of captive grey mouse lemurs [20]. Daubentonia madagascariensis herpesviridae-like sequence (DmadHVLS; scaffolded multiple fragments each <20 kb and predicted to be exogenous) was identified from data mining of a sample from a captive aye-aye [21] (sequence contigs were published as part of their supplementary material). DmadHVLS formed a sister group to members of the *Rhadinovirus* genus, a mammal-infecting genus within *Gammaherpesvirinae* [21]. A herpesvirus has also been associated with fatal encephalitis in a black-and-white ruffed lemur, although sequences are unavailable [66]. For rodents, gammaherpesvirus and betaherpesvirus genome sequences are available from wood mouse*,* guinea pig*,* common African rat*,* marmot*,* house mouse*,* steppe mouse*,* bank vole, small-eared pygmy rice rat, brown rat and black rat samples [67,74].

Here, we have characterized two coding-complete mouse lemur gammaherpesvirus sequences named lemur herpesvirus 1 which share 99.9% genome-wide identity between themselves (PX705243, 121,825 nt; PX705249, 121,961 nt), a partial lesser tufted-tailed rat betaherpesvirus genome named tufted-tailed rat herpesvirus 1 (PX705246; 151,713 nt), a partial black rat betaherpesvirus genome named black rat herpesvirus 1 (PX705242; 212,811 nt) and a partial black rat gammaherpesvirus genome named black rat herpesvirus 2 (PZ014654; 131,431 nt).

**Fig. 3.**
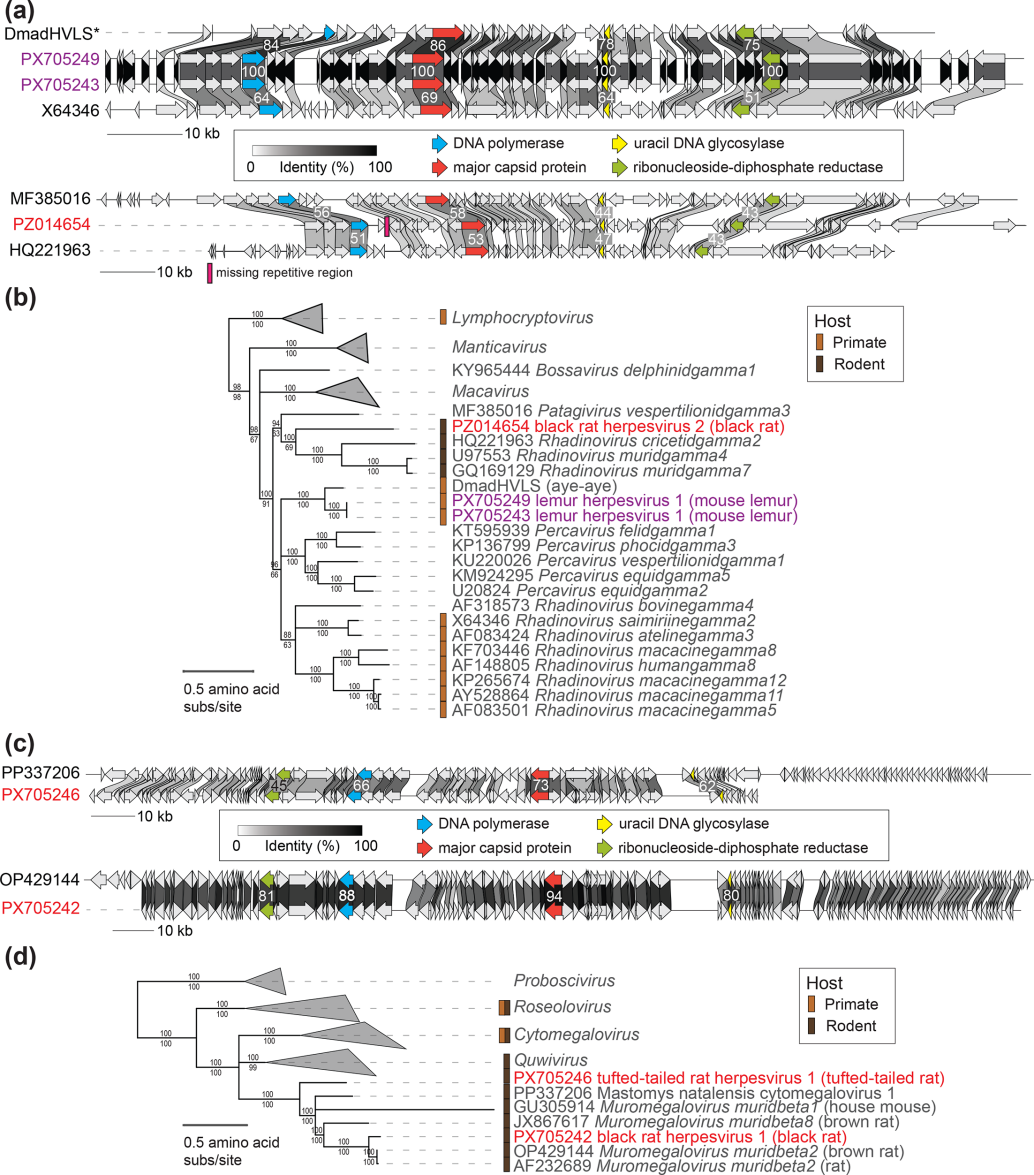
(**a**) Genome organization and protein sequence comparison of gammaherpesviruses identified in this study and their most closely related sequences. Percent amino acid identity values are shown for select proteins included in the tree. (**b**) Partitioned maximum likelihood phylogenetic tree of concatenated amino acid alignments of DNA polymerase, major capsid protein, uracil DNA glycosylase and ribonucleoside-diphosphate reductase sequences of gammaherpesviruses. Red and purple font colour signifies viral sequences identified in this study from rodents (red) and lemurs (purple). *Concatenated sequences from the supplementary material of the study by Aswad and Katzourakis [21] which we annotated and analysed. (**c**) Genome organization and protein sequence comparison of betaherpesviruses identified in this study and their most closely related sequences. Percent amino acid identity values are shown for select proteins included in the tree. (**d**) Partitioned maximum likelihood phylogenetic tree of concatenated amino acid alignments of DNA polymerase, major capsid protein, uracil DNA glycosylase and ribonucleoside-diphosphate reductase sequences of betaherpesviruses. Percent aLRT branch (above) and UFBoot (below) support are shown for each branch.

Lemur herpesvirus 1 genome sequences share highest amino acid identity for key proteins of interest (DNA polymerase, major capsid protein, uracil DNA glycosylase and ribonucleoside-diphosphate reductase) with published, partial sequences of cheirogaleid gammaherpesvirus 2 DNA polymerase (94%) (KT698106 [20]) and DmadHVLS (84%) [21] (Fig. 3a). Lemur herpesvirus 1 identified here represents the first coding-complete herpesvirus sequence from lemurs. Lemur herpesvirus 1 DNA polymerase, major capsid protein, uracil DNA glycosylase and ribonucleoside-diphosphate reductase concatenated amino acid sequences are phylogenetically positioned in a clade with those of DmadHVLS, contributing to a sister clade to the percaviruses and rhadinoviruses (Fig. 3b). Percaviruses have been characterized in equine, cat, badger, seal and bat samples. Rhadinoviruses include various disease-causing rodent, non-human primate and human gammaherpesviruses (e.g. Kaposi’s sarcoma herpesvirus). Lemur herpesvirus 1 represents a putative new species and we suggest that lemur-infecting gammaherpesviruses are likely to constitute a new genus.

Tufted-tailed rat herpesvirus 1 from this study has a genome organization similar to that of Mastomys natalensis cytomegalovirus 1 (PP337206) [72], sharing 67% identity with 28% genome coverage based on blastn, although it is missing the terminal repeat region (Fig. 3c). Tufted-tailed rat herpesvirus 1 shares highest amino acid identity of 74% for major capsid protein of murid betaherpesvirus 1 from *Mus spicilegus* (MH118555) [75] and 78% for the uracil DNA glycosylase of murid betaherpesvirus 1 from *M. musculus domesticus* (MH118557) [75]. In addition, tufted-tailed rat herpesvirus 1 DNA polymerase sequence shares the highest amino acid sequence identity of 68% with that of Mastomys natalensis cytomegalovirus 3 (OP429128) and ribonucleoside-diphosphate reductase large subunit shares 45% with that of Mastomys natalensis cytomegalovirus 1 (OP429122) identified in common African rat kidney samples [76]. Phylogenetic analysis of the DNA polymerase, major capsid protein, uracil DNA glycosylase and ribonucleoside-diphosphate reductase concatenated amino acid sequences suggests that tufted-tailed rat herpesvirus 1 falls within the *Betaherpesvirinae* subfamily and is most closely related to the rodent-infecting muromegaloviruses (Fig. 3d). Tufted-tailed rat herpesvirus 1 represents a new betaherpesvirus species and is the first herpesvirus sequence from a Nesomyinae rodent.

Black rat herpesvirus 1 identified in this study shares 84% identity with 90% genome coverage with murid betaherpesvirus 2 (OP429144) from the salivary gland of a brown rat [76]. Based on the phylogenetic analysis of DNA polymerase, major capsid protein, uracil DNA glycosylase and ribonucleoside-diphosphate reductase concatenated amino acid sequences, black rat herpesvirus 1 is a variant in the species *Muromegalovirus muridbeta2*, sharing 88% DNA polymerase protein identity with that of murid betaherpesvirus 2 (OP429144) and 94% major capsid protein identity with that of rat cytomegalovirus Maastricht (AF232689) [77], (Fig. 3c, d).

Black rat herpesvirus 2 is a gammaherpesvirus which shares 68% identity with just 8% genome coverage with Equid gammaherpesvirus 2 (MW322583; a member of *Percavirus equidgamma2*). However, for core genes of interest, black rat herpesvirus 2 shares 56% DNA polymerase amino acid identity and 58% major capsid protein amino acid identity with vespertilionid gammaherpesvirus 3 (MF385016) from the lung of a big brown bat. Black rat herpesvirus 2 DNA polymerase, major capsid protein, uracil DNA glycosylase and ribonucleoside-diphosphate reductase concatenated amino acid sequences are phylogenetically positioned in a clade with those of viruses representing *Patagivirus vespertilionidgamma3* (MF385016, big brown bat-associated), *Rhadinovirus cricetidgamma2* (HQ221963, pygmy rice rat-associated), *Rhadinovirus muridgamma4* (U97553, house mouse-associated) and *Rhadinovirus muridgamma7* (GQ169129, wood mouse-associated). We suggest that the taxonomic classification of *Rhadinovirus cricetidgamma2*, *Rhadinovirus muridgamma4* and *Rhadinovirus muridgamma7* as rhadinoviruses may need to be revisited given the phylogenetic information gained from black rat herpesvirus 2.

Lemur herpesvirus 1 identified here, together with the DmadHVL sequences [21], supports a lemur-specific lineage of gammaherpesvirus that is distantly related to primate-infecting rhadinovirids. On the other hand, tufted-tailed rat herpesvirus 1, the first from a Nesomyinae rodent, shows an evolutionary relationship with other muromegalovirids including black rat herpesvirus 1, a variant and member of the *Muromegalovirus muridbeta2* species, demonstrating the movement and maintenance of rat herpesviruses across geographic regions. Additionally, black rat herpesvirus 2 displays significant genetic diversity, contrasting with the primary findings on black rat-infecting viruses in this study, contributing to a lineage of distantly related bat- and rodent-infecting gammaherpesviruses.

### Papillomaviridae

The *Papillomaviridae* family is composed of viruses with icosahedral virions that encapsidate circular dsDNA genomes ranging in size from ~6 to 8 kb [78]. PVs are generally species-specific with tropism for cutaneous and mucosal epithelia. The majority of PVs persist asymptomatically in latent, long-term infections. However, some PVs have been found to cause a range of symptoms, from benign warts (i.e. papillomas) to cervical, lung and head-and-neck cancers [79,81]. *Papillomaviridae* is an expansive family whose members are found to infect various mammals, birds, reptiles and fish [78]. PV genomes encode for late proteins, including the minor capsid protein (L2) and highly conserved major capsid protein (L1), along with early gene products associated with viral replication and maintenance (E1–E6). The species demarcation threshold for PVs is 70% L1 nucleotide identity, while PV types within a species are differentiated by 10% nucleotide dissimilarity from known PVs [82,83]. Three PV types, Varecia variegata papillomavirus type 1, Varecia variegata papillomavirus type 2 (VavPV2) and Varecia rubra papillomavirus type 1, have been identified from captive lemurs [7,19]. These three previously published PV types form a distinct lineage, likely representing a new genus in the *Papillomaviridae* family. Similar to the PVs of numerous mammalian groups, rodent PVs are not monophyletic, falling into multiple lineages (i.e. *Pipapillomavirus*, *Iotapapillomavirus*, *Sigmapapillomavirus* and *Dyosigmapapillomavirus* genera) [84]. Rodent PVs have primarily been identified as asymptomatic or associated with lesions such as papillomas and keratoacanthomas [85,86].

In this study, we identified five complete PV genomes from lemurs: two from black-and-white ruffed lemurs (PX705260, 7,721 nt in length; PX705257, 7,715 nt), one from a mouse lemur (PX705262, 7,643 nt) and two from woolly lemurs (PX705250, 7,181 nt; PX705254, 7,513 nt). In addition, we also identified four complete PV genomes from black rats (PX705253, 7,777 nt; PX705256, 7,631 nt; PX705259, 7,363 nt; and PX705263, 7,780 nt).

**Fig. 4.**
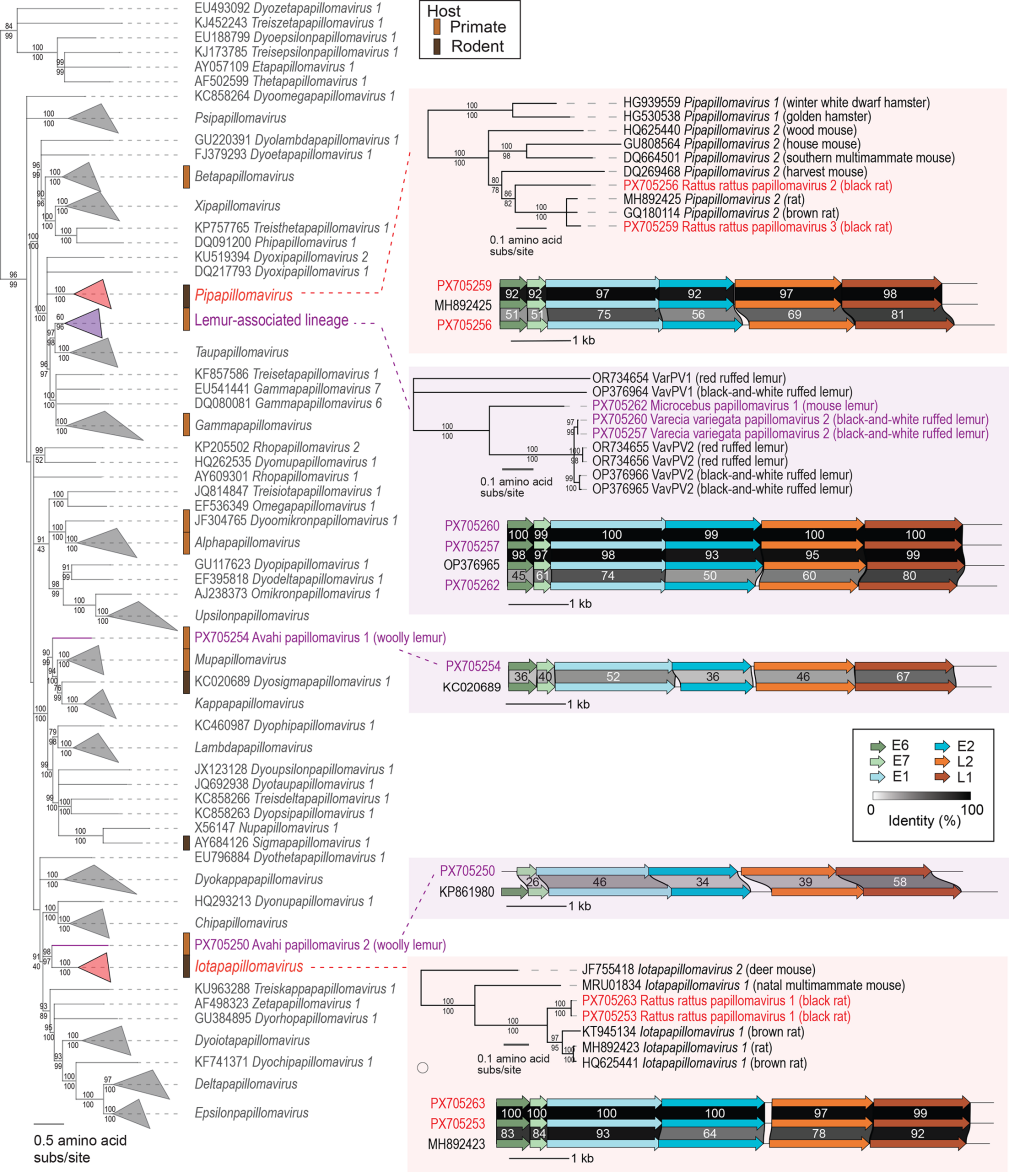
Partitioned maximum likelihood phylogenetic tree of concatenated amino acid alignments of E1, E2 and L1 with representative members of established PV species, captive lemur PVs and PVs identified in this study. Red and purple font colour signifies viral sequences identified in this study from rodents (red) and lemurs (purple). Genome organization and protein sequence comparison of PVs identified in this study and their most closely related sequences. Percent aLRT branch (above) and UFBoot (below) support are shown for each branch.

PV genomes identified from black-and-white ruffed lemur oral swabs, PX705260 and PX705257, are members of VavPV2 type, given they share 96.9% genome-wide sequence identity to representative genomes of VavPV2 previously identified in captive black-and-white ruffed lemurs at the Duke Lemur Center (Durham, NC, USA) [7,19]. The VavPV2 sequences identified here share 100% L1 nucleotide identity to each other and 97.7% to VavPV2 isolate D24SS (OP376965) [19] (Sheet S3, Supplementary Material 2). This finding demonstrates VavPV2’s long-term persistence in black-and-white ruffed lemurs in both natural and captive settings. One PV genome (PX705262) was identified in a mouse lemur oral swab and shares 71.3% L1 nucleotide identity and 66.3% genome-wide nucleotide identity with VavPV2 isolate D24SS (OP376965) (Sheet S3, Supplementary Material 2). Thus, this genome represents a novel PV type named Microcebus papillomavirus 1 and, together with VavPV2, represents a unique new species. These three PV genomes add to the growing number of identified sequences with this lemur PV lineage (Fig. 4). On the other hand, the two woolly lemur PV genomes identified in this study (sharing 48% genome-wide pairwise identity to each other) are significantly divergent from other lemur PVs phylogenetically (based on the concatenated amino acid sequence analysis of the E1, E2 and L1 proteins) and form two distinct lemur PV lineages. This now indicates we have three lemur-PV lineages (Fig. 4). One of the woolly lemur PVs (PX705254) shares highest similarity, 68% with 38% genome coverage (based on blastn analysis) identity and 66.0% L1 nucleotide identity, with the sequence of Castor canadensis papillomavirus type 1 (KC020689), a North American beaver PV classified in the species *Dyosigmapapillomavirus 1* [87] (Sheet S3, Supplementary Material 2). The E1, E2 and L1 protein sequences of this PV sit basal to those of the members of the *Mupapillomavirus* (human-associated), *Kappapapillomavirus* (lagomorph-associated) and *Dyosigmapapillomavirus* (rodent-associated) genera (Fig. 4). This PV represents a putative new species and type and has been named Avahi papillomavirus 1. It is important to note that the genome of Avahi PV 1 is missing the E6 gene, while its related sequences do have an intact E6 (Fig. 4). The absence of E6 in PVs may influence viral replication or persistence strategies and has been noted in a variety of PV genera including *Epsilonpapillomavirus*, *Etapapillomavirus*, *Thetapapillomavirus*, *Xipapillomavirus* and *Zetapapillomavirus* [78,82]. The second woolly lemur-derived PV (PX705250) shares 66% identity with 32% genome coverage (based on Blastn analysis) and 64% L1 nucleotide identity with the sequence of Alouatta guariba papillomavirus 1 (KP861980) from a southern brown howler monkey [88] classified in the species *Dyoomikronpapillomavirus 1* (Sheet S3, Supplementary Material 2). This woolly lemur PV also represents a putative new species and type named Avahi papillomavirus 2, whose E1, E2 and L1 proteins phylogenetically fall basal to the rodent-infecting PVs in the *Iotapapillomavirus* genus (Fig. 4). The phylogenetic positions of Avahi PVs in relation to the other lemur PVs suggest that there are multiple lineages of PVs in lemurs and in particular the woolly lemurs.

Two PV genomes (PX705253 and PX705263) were identified from black rat oral swabs. These PVs share 99.7% genome-wide sequence similarity and 99.7% L1 nucleotide identity with one another along with 77% genome-wide sequence similarity with 100% genome coverage (based on Blastn analysis) and 81.6% L1 nucleotide identity with the sequence of Rattus norvegicus papillomavirus 2 (MH892423) from a wild rat skin sample from China [89] which is classified in the species *Iotapapillomavirus 1*. These PVs thus constitute a new PV type in species *Iotapapillomavirus 1*, which we have named Rattus rattus papillomavirus 1. Other PVs which are part of the genus *Iotapapillomavirus* have been previously identified from brown rat, deer mouse, natal multimammate mouse and *Rattus* sp. faeces, oronasopharynx and skin samples from China, Denmark, Germany and the USA [90,93].

Two additional PVs (PX705256 and PX705259) from black rats, sharing 72.4% L1 nucleotide identity and 69.4% genome-wide sequence similarity with each other, were identified. These two share 72.4 and 88.7% L1 nucleotide identity (70.0% identity with 81% genome coverage and 92.0% identity with 100% genome coverage based on blastn analysis), respectively, with the sequence of Papillomaviridae RPVne-OR02-zj (MH892425), a member of *Pipapillomavirus 2*, from a wild rat oral swab from China [89]. These two represent two new PV types which we have named Rattus rattus papillomavirus 2 (PX705256) and 3 (PX705259) and are members of the species *Pipapillomavirus 2*. Thus, members of *Pipapillomavirus 2* have been identified in the wood mouse*,* southern multimammate mouse*,* Eurasian harvest mouse, house mouse, brown rat and, newly, the black rat [93,96].

Animal PVs exhibit long-standing co-evolutionary relationships with their hosts; for example, PVs are estimated to have been circulating in rodents for a minimum of 17 million years [97]. The discovery of iotapapillomaviruses and pipapillomaviruses in black rats in Madagascar shows the maintenance of PVs in rat populations. Our work reflects an evolutionary history of PVs in mouse lemurs (family Cheirogaleidae) and black-and-white ruffed lemurs (family Lemuridae), distantly related lemur lineages, which appears to differ from the two woolly lemur lineages.

### Polyomaviridae

PyVs have been found to primarily asymptomatically infect mammals, birds and fish, although symptomatic infection or cancer has been observed in immunocompromised hosts (e.g. Merkel cell PyV’s association with Merkel cell carcinoma in humans) [98]. Viruses in the *Polyomaviridae* family have icosahedral virions with circular dsDNA genomes ranging in size from 4 to 7 kb in length [98]. PyV genomes usually encode for two regulatory proteins (large and small tumour antigen proteins) and three capsid proteins (VP1, VP2 and VP3) with their oncogenic potential highly dependent on the large T antigen protein (LTag) [98,99]. The *Polyomaviridae* family is composed of six genera: *Alphapolyomavirus*, *Betapolyomavirus*, *Gammapolyomavirus*, *Deltapolyomavirus, Epsilonpolyomavirus* and *Zetapolyomavirus*. The PyV species demarcation threshold is 85% large T-antigen nucleotide identity with that of the most closely related PyV species coupled with host specificity and tissue tropism [98].

PyVs have largely been shown to be host-specific, indicating virus–host co-divergence [100]. However, studies have revealed a complex bat PyV evolutionary history, suggesting host-jumping and emphasizing the importance of studying PyVs across diverse mammalian taxa [101,102]. Decades of research on non-human primate and rodent PyVs (e.g. simian virus 40 and murine PyV), in particular, have driven our understanding of PyV biology [103]. While PyVs have been identified in great apes, Old World Monkeys and New World Monkeys [104,105], the strepsirrhine lineage – lemurs, lorises, galagos and pottos – had been poorly sampled for PyVs, with no PyV sequences determined [104]. Primate-infecting PyVs have been found to cluster in primate-specific clades spread across the phylogenetic tree of sequences of members of the *Polyomaviridae* family.

**Fig. 5.**
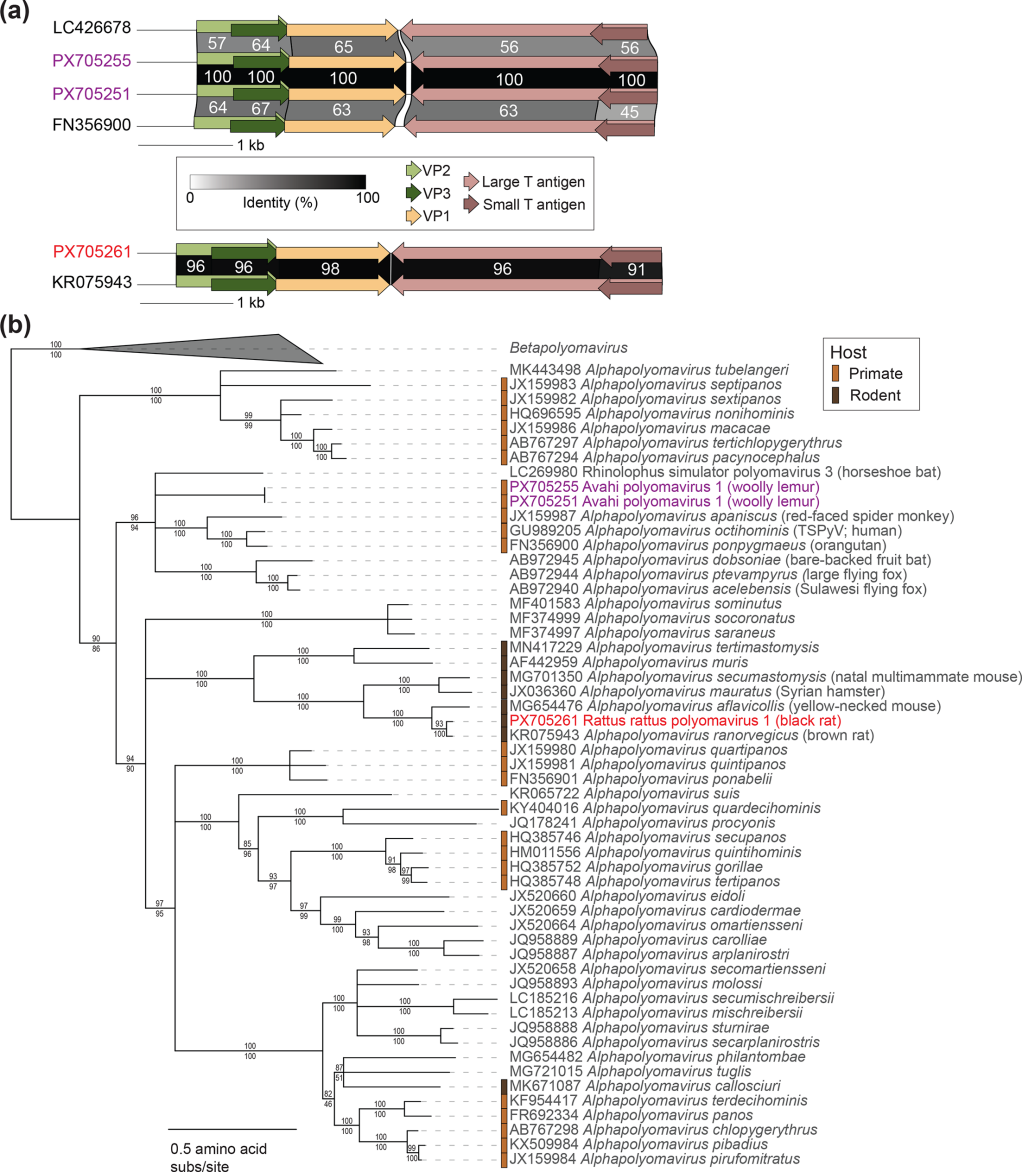
(**a**) Genome organization and protein sequence comparison of PyVs identified in this study and their most closely related sequences. (**b**) Maximum likelihood phylogenetic tree of large T-antigen amino acid sequences of representative viruses from established *Alphapolyomavirus* species and of those identified in this study. Red and purple text font colour signifies viral sequences identified in this study from rodents (red) and lemurs (purple). Percent aLRT branch (above) and UFBoot (below) support are shown for each branch.

Two PyV genomes (PX705251, 5,197 nt; PX705255, 5,198 nt) were identified from woolly lemurs and share 99.9% genome-wide sequence similarity and 100% LTag amino acid identity with one another (Fig. 5a, Sheet S4, Supplementary Material 2). These two PyVs have been named Avahi polyomavirus 1. Avahi PyV 1 sequences share 67% identity with 79% genome coverage based on Blastn analysis with genome-wide sequence similarity with Bornean orangutan polyomavirus (FN356900) [106]. As Avahi PyV 1 sequences share the highest *LTag* nucleotide identity, 67.5%, with Rhinolophus simulator polyomavirus 3 from horseshoe bats (LC269980) [107], Avahi PyV 1 represents a novel PyV species (Sheet S4, Supplementary Material 2). Avahi PyV 1 LTag sequences display a phylogenetic relationship with a clade of primate PyVs including members of *Alphapolyomavirus apaniscus* from spider monkeys [104], *Alphapolyomavirus octihominis* [i.e. Trichodysplasia spinulosa-associated polyomavirus (TSPyV)] from humans [108] and *Alphapolyomavirus ponpygmaeus* from orangutans [106] (Fig. 5a, b). TSPyV has been identified as the causative agent of trichodysplasia spinulosa, a rare skin disease in humans impacting immunocompromised patients [108,109]. Avahi PyV 1 also shares an evolutionary history with bat PyVs including Rhinolophus simulator polyomavirus 3 (LC269980) and members of *Alphapolyomavirus dobsoniae*, *Alphapolyomavirus ptevampyrus* and *Alphapolyomavirus acelebensis* [107,110] (Fig. 5a, b). Bat PyV lineages fall across the *Polyomaviridae* tree, showing likely historical PyV interactions between bats and multiple mammalian orders, including non-human primates, through evolutionary time [101]. Avahi PyV 1 represents the first PyV from strepsirrhine primates and contributes a diverse PyV in a lineage with a human disease-associated PyV.

A PyV genome (PX705261, 5,312 nt in length) was identified from a black rat and has been named Rattus rattus polyomavirus 1. Rattus rattus PyV 1 shares 89.8% *LTag* nucleotide identity and 91.7% genome-wide sequence similarity with Rattus norvegicus PyV 1 isolate 3,687 (KR065724), a sequence of *Alphapolyomavirus ranorvegicus* characterized from a brown rat [59,111]. Thus, Rattus rattus PyV 1 is a member of *Alphapolyomaviru*s *ranorvegicus* infecting black and brown rats, two rat species which have demonstrated incredible adaptation to anthropogenic spaces across the planet (Fig. 5b). *Alphapolyomavirus ranorvegicus* had been characterized from brown rat spleen, kidney and abdominal cavity samples from Germany [111], Iran and Hungary [112] and Tanezumi rat lung samples from China [59]. Our research expands the known geographic, biogeographic and host species range of *Alphapolyomavirus ranorvegicus*.

### Parvoviridae

The family *Parvoviridae* has three subfamilies, *Densovirinae*, *Hamaparvovirinae* and *Parvovirinae*. Viruses in the *Parvovirinae* subfamily have been found to infect mammals, birds and reptiles with wide variation in pathogenicity [113]. Their linear, ssDNA genomes are 4–6 kb in length encoding for replication initiator (NS1) and capsid (VP) proteins – with some encoding additional proteins, e.g. NP1 in *Bocaparvovirus* [114] – and terminating in boxed hairpin structures [113]. Within the subfamily *Parvovirinae,* viruses in the genus *Dependoparvovirus*, i.e. adeno-associated viruses (AAVs), are latent, defective viruses replicating only in cells co-infected with a helper DNA virus, generally an adenovirus [113]. Despite the potential applications of non-human primate AAVs in improving human health as gene therapy vectors [115], the only non-human primate-infecting dependoparvovirus sequences available are from macaques [116,117], spider monkeys and white-fronted capuchins. On the other hand, for animals in the order Rodentia, dependoparvovirus sequences have been characterized from woodland dormouse, long-tailed marmot, Mongolian marmot, Himalayan marmot, house mouse, nutria, bank vole, deer mouse, brown rat and Tanezumi rat samples [59,62, 90, 118,123].

A few viruses in the *Bocaparvovirus* genus (*Parvovirinae* subfamily) are thought to be associated with widespread disease outbreaks in cattle (bovine parvovirus 1; gastrointestinal and respiratory disease, reproductive failure and conjunctivitis) and dogs (minute virus of canines; enteritis in adults, neonatal respiratory disease or abortion in foetuses) [113]. Human bocavirus 1 may be associated with lower respiratory infections in children, particularly with other viral co-infections [124]. However, as more bocaparvoviruses are being discovered, they are thought to be widespread across mammalian clades with limited known pathogenicity. For viruses in the genera *Dependoparvovirus* and *Bocaparvovirus*, the species demarcation threshold is 85% based on NS1 amino acid identity [113,125].

**Fig. 6.**
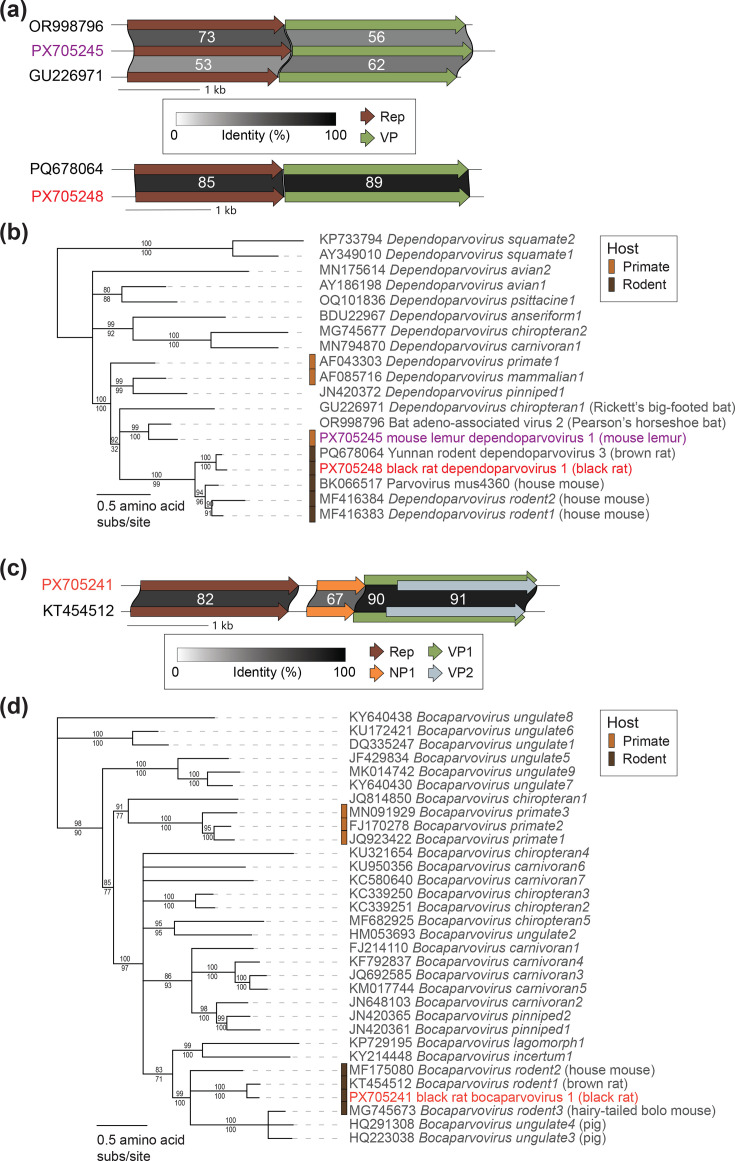
(**a**) Genome organization and protein sequence comparison of dependoparvoviruses identified in this study and their most closely related sequences. (**b**) Maximum likelihood phylogenetic tree of NS1 amino acid sequences of representative sequences of established species in the genus *Dependoparvovirus* and sequences identified in this study. The phylogenetic tree is rooted with bocaparvovirus sequences. Red and purple font colour signifies viral sequences identified in this study from rodents (red) and lemurs (purple). (**c**) Genome organization and protein sequence comparison of bocaparvoviruses identified in this study and their most closely related sequences. (**d**) Maximum likelihood phylogenetic tree of NS1 amino acid sequences of representative sequences of established species in the genus *Bocaparvovirus* and sequences identified in this study. The phylogenetic tree is rooted with dependoparvovirus sequences. Red font colour signifies rodent-associated viral sequences identified in this study. Percent aLRT branch (above) and UFBoot (below) support are shown for each branch.

In this study, we present sequences of two dependoparvoviruses, one from a mouse lemur oral swab named mouse lemur dependoparvovirus 1 (PX705245, 4,704 nt) and one from a black rat oral swab named black rat dependoparvovirus 1 (PX705248, 4,353 nt). The mouse lemur dependoparvovirus 1 shares 74% identity with 73% genome coverage (based on Blastn analysis) and 73% NS1 amino acid identity with that of bat AAV 1 (OR998796) [126] from a Pearson’s horseshoe bat faecal swab and represents a new species (Fig. 6a). Mouse lemur dependoparvovirus 1 together with bat AAV 1 are most closely related to representative NS1 sequence from the *Dependoparvovirus chiropteran1* species but are not monophyletic with them (Fig. 6b). Mouse lemur dependoparvovirus 1 is the first dependoparvovirus discovered in strepsirrhine primates.

Black rat dependoparvovirus 1 shares 88% identity with 97% genome coverage (based on Blastn analysis) and 89.7% NS1 amino acid identity with the sequence of Yunnan rodent dependoparvovirus 3 (PQ678064) from a brown rat gut sample [59] (Fig. 6c). Black rat dependoparvovirus 1 has a close phylogenetic relationship with a clade of rodent-associated dependoparvoviruses (Fig. 6d). Black rat dependoparvovirus 1 and Yunnan rodent dependoparvovirus 3 represent a new dependoparvovirus species.

One bocaparvovirus genome encoding a Rep (NS1), VP1, VP2 and NP1 was identified from a black rat oral swab (PX705241, 5,396 nt) and named black rat bocaparvovirus 1. Black rat bocaparvovirus 1 shares 80% identity with 98% genome coverage (blastn analysis) and 82% Rep amino acid identity with the sequence of rat bocavirus strain HK1S (KT454512) from a brown rat from Hong Kong [127] (Fig. 6d). Black rat bocaparvovirus 1 represents a new species with a close evolutionary relationship to viruses in the species *Bocaparvovirus rodent1* from brown rats (Fig. 6c). The close phylogenetic relationship between black rat bocaparvovirus 1 and viruses in the species *Bocaparvovirus rodent1* suggests potential virus–host co-evolution for closely related *Rattus* species hosts (*R. rattus*, *R. norvegicus*).

### Circoviridae

Viruses in the *Circoviridae* family within the *Cressdnaviricota* phylum have circular ssDNA genomes encoding two major proteins, the replication-associated protein (Rep) and capsid protein (Cp) with ambisense genome organization [128]. *Circoviridae* is composed of two genera, *Circovirus* and *Cyclovirus* [129,130]. Circoviruses, viruses in the genus *Circovirus*, have been characterized from diverse mammal, bird and fish species with some circoviruses associated with severe disease (e.g. porcine circovirus type 2 and beak and feather disease virus) [129]. For primates, circoviruses have recently been identified in the samples of humans [131,133]. For rodents, complete circovirus genomes have been characterized from the samples of house mice, Clarke’s voles, Chevrier’s field mice, brown rats, South China field mice, smoke-bellied rats, bamboo rats, Mongolian five-toed jerboas and Merriam’s kangaroo rats [31,134, 135]. The species demarcation threshold for circoviruses is 80% genome-wide nucleotide sequence identity [129,130].

**Fig. 7.**
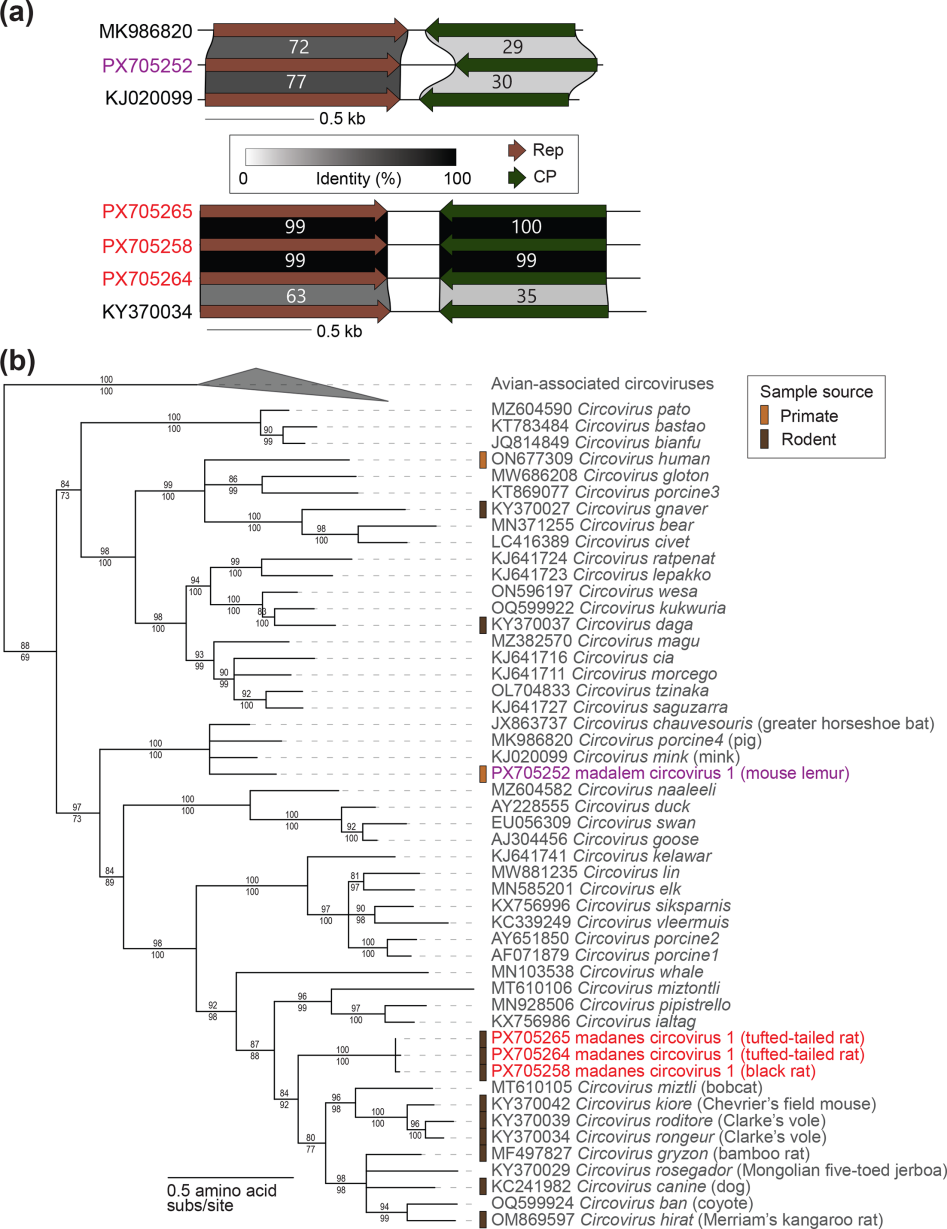
(**a**) Genome organization and protein sequence comparison of circoviruses identified in this study and their most closely. (**b**) Maximum phylogenetic tree of Rep amino acid sequences of established species in *Circovirus* and circovirus sequences identified in this study. Font colour signifies viral sequences identified in this study from rodents, red, or lemurs, purple. Percent aLRT branch (above) and UFBoot (below) support are shown for each branch.

Four complete circovirus genomes were identified in this study from mouse lemur, Webb’s tufted-tailed rat and black rat oral swabs. These genomes exhibit typical circovirus genome organization, encoding for Cp and Rep (Fig. 7a). The circovirus genome identified from a mouse lemur oral swab (PX705252, 1,862 nt) has been named madalem circovirus 1 (name derived from **Mada**gascar **lem**ur). Madalem circovirus 1 shares 60.5–63.8% genome-wide nucleotide sequence identity with representative virus genomes in the established species *Circovirus mink* (KJ020099) [136]*, Circovirus porcine4* (MK986820) [137] and *Circovirus chauvesouris* (JX863737) [138] (Sheet S5, Supplementary Material 2). The Rep of Madalem circovirus one also clusters with representative sequences from these species (Fig. 7b). Madalem circovirus one represents a putative novel circovirus species falling within a lineage of mammal-infecting circoviruses in diverse hosts (i.e. mink, pig and greater horseshoe bat) (Fig. 7a). While the pathogenicity of viruses in the species *Circovirus chauvesouris* characterized from bats remains unknown [138], those of *Circovirus mink* and *Circovirus porcine4* have both been shown to be pathogenic to their hosts [136,139]. The adult mouse lemur sampled appeared healthy, although further investigation is necessary to determine the pathogenic and cross-species potential of the first circovirus identified from the lemuriform primates.

Three circovirus genomes were identified from the oral swabs of two Webb’s tufted-tailed rats (PX705264 and PX705265, 2,105 nt) and one black rat (PX705258, 2,105 nt), and these three share 97.0–98.1% genome-wide similarity with one another (Sheet S5, Supplementary Material 2). This virus has been named madanes circovirus 1 (name derived from **Mada**gascar **Nes**omyinae). Madanes circovirus 1 genomes share 60% genome-wide nucleotide identity with rodent-associated circovirus 1 isolate RtMc-CV-1/Tibet2014 (KY370034 [31]) from Clarke’s voles in Tibet (Sheet S5, Supplementary Material 2). Thus, madanes circovirus 1 represents a novel circovirus species and denotes the possibility of either interspecies circovirus infection between non-native and endemic rodents or detection as a result of coprophagy of another rodent faeces where shed circoviruses can be present. Phylogenetic analysis of the Rep amino acid sequences places madanes circovirus 1 in a lineage of primarily rodent-associated circoviruses from Clarke’s vole, Chevrier’s field mouse, bamboo rat, Mongolian five-toed jerboa and Merriam’s kangaroo rat samples along with predator-associated circoviruses from bobcat, dog and coyote samples [31,134, 135, 140, 141] (Fig. 7a). As the home ranges of tufted-tailed rats and black rats overlap in the lowland rainforest of MSR creating competition for limited resources, close contact and potential conflict between black rats and Webb’s tufted-tailed rats or faecal consumption (coprophagy or through environmental contamination) could lead to opportunities for interspecies circovirus infection.

### Detection frequency and co-infections

**Fig. 8.**
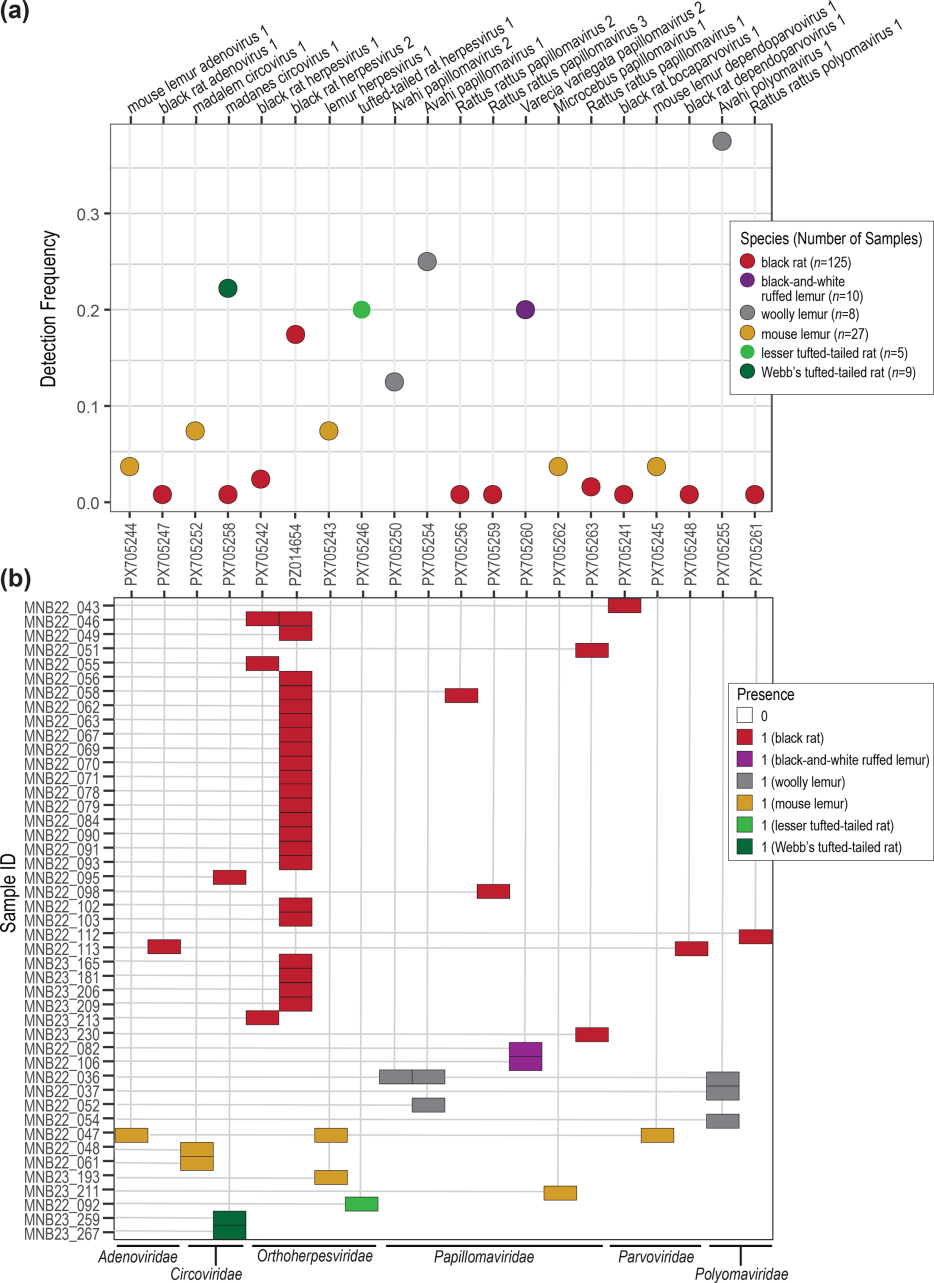
(**a**) Detection frequency of vOTUs identified in this study for each species sampled. (**b**) Viral co-infections within individuals were identified for woolly lemurs, mouse lemurs and black rats. vOTU presence is defined as ≥75% sequence coverage. Samples with at least one vOTU present were included in this heatmap to confirm co-infections.

The 26 virus sequences were clustered at 95% ANI into 20 vOTUs for the determination of detection frequency and co-infections. We found low frequency of detection (<8%) for adenoviruses, herpesviruses and parvoviruses across the dataset with the exception of black rat herpesvirus 2 which had a detection frequency of 17.6% in black rats (Fig. 8a, Sheets S6 and S7, Supplementary Material 2). Given the larger sample size for black rats and the detection of black rat herpesvirus 2 in multiple individuals, for black rats where we were able to determine age class, black rat herpesvirus 2 was detected in 36.4% (20/55) of adult samples and 3.3% (2/60) of juvenile/subadult samples. Higher detection frequency in adults may suggest exposure over time and the establishment of herpesvirus infections persisting through adulthood. Black rat herpesvirus 2 detection frequency also differed across sampling years with detection in 29.0% (18/62) of 2022 samples and 6.3% (4/63) of 2023 samples, potentially reflecting higher sample numbers of juveniles/subadults (*n*=39) for 2023 in comparison with 2022 (*n*=21). Black rat herpesvirus 2 was detected in 20.3% (13/64) of male samples and 14.8% (9/61) of female samples. For black rats, most viruses identified were present in only one to two individuals, highlighting the importance of sample size particularly for rodents with large, fluctuating populations compared to the sample size required for heavily declining lemur populations at the MSR. Larger-bodied lemur populations, such as the critically endangered black-and-white ruffed lemurs, have experienced alarming declines and near decimation in many regions including the MSR, whereas small-bodied, generalist cheirogaleid lemurs are thought to be more resilient throughout anthropogenic landscapes, showing comparatively less decline [142,144]. For lemur-associated PVs and PyVs, some had higher detection frequency including 37.5% for Avahi polyomavirus 1 in woolly lemurs and 20% for VavPV2 in black-and-white ruffed lemurs, whereas Microcebus papillomavirus 1 had a detection frequency of 3.7% in mouse lemurs (Fig. 8a, Sheet S7, Supplementary Material 2). We suggest that higher lemur-associated virus detection frequency reflects both that we have sampled a larger proportion of the population in the region and that larger-bodied lemur individuals are likely frequently interacting and highly connected within their small, isolated populations at the MSR, allowing for ease of virus sharing and maintenance. PV detection in non-human primates has been found to vary widely with both sample type and PV genus potentially playing a role; for example, rhesus macaque oral swabs were found to have 7.1% alphapapillomavirus and 9.5% betapapillomavirus prevalence yet 90.5% gammapapillomavirus prevalence [145]. Further, primate skin samples have shown 75–87% PV prevalence, inclusive of all PV types examined in that study, for chimpanzees, gorillas and long-tailed macaques [146]. PyV prevalence in non-human primates has additionally been found to differ by sample type, with organ and skin samples demonstrating higher prevalence (7–43%) than faecal and blood samples (<4%) [104]. Thus, we would similarly expect varying detection frequencies for lemur-associated PVs and PyVs for different lineages and sample types.

As our threshold for vOTU presence/absence in a sample was 75% genome coverage, detection frequencies for larger viruses such as lemur herpesvirus 1 (genome size 122 kb) may be underestimated. For example, with a 50% coverage cut-off for vOTU presence (67.2% coverage at 98.1% identity for one individual, 52.8% coverage at 99.0% identity for a second individual), lemur herpesvirus 1 has a detection frequency of 14.8%. However, the use of a 50% coverage threshold provides lower confidence, as more incomplete genome coverage is more likely to omit key regions that may have high sequence variability.

A few cases of co-infections were seen throughout the study samples (Fig. 8b). As AAV replication occurs only in cells co-infected with usually an adenovirus or herpesvirus [113], interesting co-infection cases described in this study include the identification of AAVs with their potential helper viruses. One mouse lemur (sample ID MNB22_047) was co-infected with mouse lemur dependoparvovirus 1 along with mouse lemur adenovirus 1, a variant in the species *Mastadenovirus lamiae*, and lemur herpesvirus 1, a mouse lemur gammaherpesvirus (Fig. 8b). While adenoviruses (e.g. adenovirus type 5 in humans) are primarily the helper viruses for AAVs, herpesviruses (e.g. herpes simplex virus types 1 and 2 and human herpesvirus 6 in humans) have also been found to serve as AAV helper viruses [147,149]. One black rat (sample ID MNB22_113) was co-infected with black rat dependoparvovirus 1 and black rat adenovirus 1 (Fig. 8b). It is highly likely that black rat adenovirus 1 is the helper virus for black rat dependoparvovirus 1. Another case of viral co-infection characterized in this study includes the infection in a woolly lemur (sample ID MNB22_036) with Avahi papillomavirus 1 and 2 and Avahi polyomavirus 1. PVs and PyVs are recognized as primarily species-specific and tissue-specific oncogenic viruses exacting lifelong infection with similarities in both structure and function. PVs and PyVs, while often asymptomatic, are both associated with various cancers, and the role of PV–PyV co-infection in cancer initiation and progression has been investigated for a select few human viruses of interest (e.g. HPV and BKV) [150]. Thus, this dataset provides evidence of co-infections – AAV–adenovirus and PV–PyV – heavily investigated in humans and rarely successfully documented in rodent and non-human primate lineages.

## Conclusion

The results presented here support the notion that the unique evolutionary history of lemurs on Madagascar has moulded distinct mammal-infecting DNA virus communities, demonstrating likely co-evolution with their geographically isolated, diverse hosts. We identified lemur-infecting viruses in the families *Adenoviridae*, *Papillomaviridae*, *Parvoviridae*, *Polyomaviridae*, *Orthoherpesviridae* and *Circoviridae*. All established primate-infecting adenovirus species fall into one large lineage except for the divergent lemur adenoviruses. Lemur PVs share exceedingly low similarity with any other primate PVs, instead forming distinct phylogenetic clades (putative novel genera) across the *Papillomaviridae* phylogeny (E1, E2 and L1 protein). The lemur AAV from this study, which represents the first identified one in lemurs, shares highest similarity with bat AAVs, falling into a lineage considered to be the origin of mammalian AAVs. The lemur PyV from this study, which represents the first identified in lemurs, is positioned with a lineage of primate-infecting PyVs which may share an evolutionary history with bat PyVs. Further, the mouse lemur gammaherpesvirus identified in this study is highly divergent from established herpesvirus species, forming a putative new genus with an aye-aye gammaherpesvirus distantly related to primate-infecting rhadinoviruses. The lemur circovirus identified here, madalem circovirus 1, represents not only the first circovirus from lemurs but the first from non-human primates. Across lemur-associated sequences of all viral families examined in this study, lemur-infecting DNA viruses are exceedingly diverse and unique, showing limited genetic similarity to other primate-infecting DNA viruses, yet in several cases retaining phylogenetic relationships with other primate viruses. These patterns emphasize the impacts of long-term geographic isolation for viral diversity and virus–host co-evolution in island ecosystems. Endemic and non-native rodent viral sequences – adenovirus, herpesvirus, PV, parvovirus, PyV and circovirus – generally showed high similarity to known rodent-infecting viral lineages, although the tufted-tailed rat betaherpesvirus and black rat gammaherpesvirus presented unique viral diversity.

As viral research in natural populations of lemurs and rodents in Madagascar has been remarkably limited despite the island’s rich evolutionary history and escalating anthropogenic pressures, this study provides a crucial genomic and phylogenetic foundation for DNA viruses in Malagasy lemurs and rodents. This work builds upon our prior work in captive populations, particularly expanding on our understanding of PV diversity in lemur populations and documenting the same PV types circulating in captive and wild individuals of the same species. We additionally highlight the utility of virus discovery for uncovering interactions between endemic and non-native host species. Madanes circovirus 1 was present in two tufted-tailed rats and one black rat, with genomes sharing 97–98% similarity, suggesting either interspecies circovirus infection or coprophagy of another rodent’s faeces which could have further consequences. As we develop a baseline catalogue of viral communities in Madagascar’s wildlife, we expect that endemic animal health and viral transmission are likely influenced by anthropogenic change, including competition and interactions with non-native species. Overall, our study works to augment incredibly sparse genomic resources for DNA viruses in endemic non-human primate and rodent populations in Madagascar, uncovering a landscape of DNA viral diversity in multiple mammalian species.

## Supplementary material

10.1099/mgen.0.001728 Supplementary Material 1.

10.1099/mgen.0.001728 Supplementary Material 2.

## Abbreviations

AAI: average amino acid identity
AAV: adeno-associated virus
aLRT: approximate likelihood ratio test
DmadHVLS: Daubentonia madagascariensis herpesviridae-like sequence
HMM: hidden Markov model
LTag: large tumour antigen
MSR: Manombo Special Reserve
PV: papillomavirus
TSPyV: Trichodysplasia spinulosa-associated polyomavirus
UFBoot: ultrafast bootstrap approximation
VavPV2: Varecia variegata papillomavirus type 2
vOTU: virus operational taxonomic unit
