# Supplementary material for: Mammal-infecting DNA viruses identified in lemurs and rodents in Madagascar mirror the evolutionary history of their hosts
Source: Microb Genom. 2026 May 22;12(5):001728. doi: 10.1099/mgen.0.001728 (PMC13196890; doi:10.1099/mgen.0.001728)
Supplement: Supplementary Material 1. [file mgen-12-01728-s001.pdf]

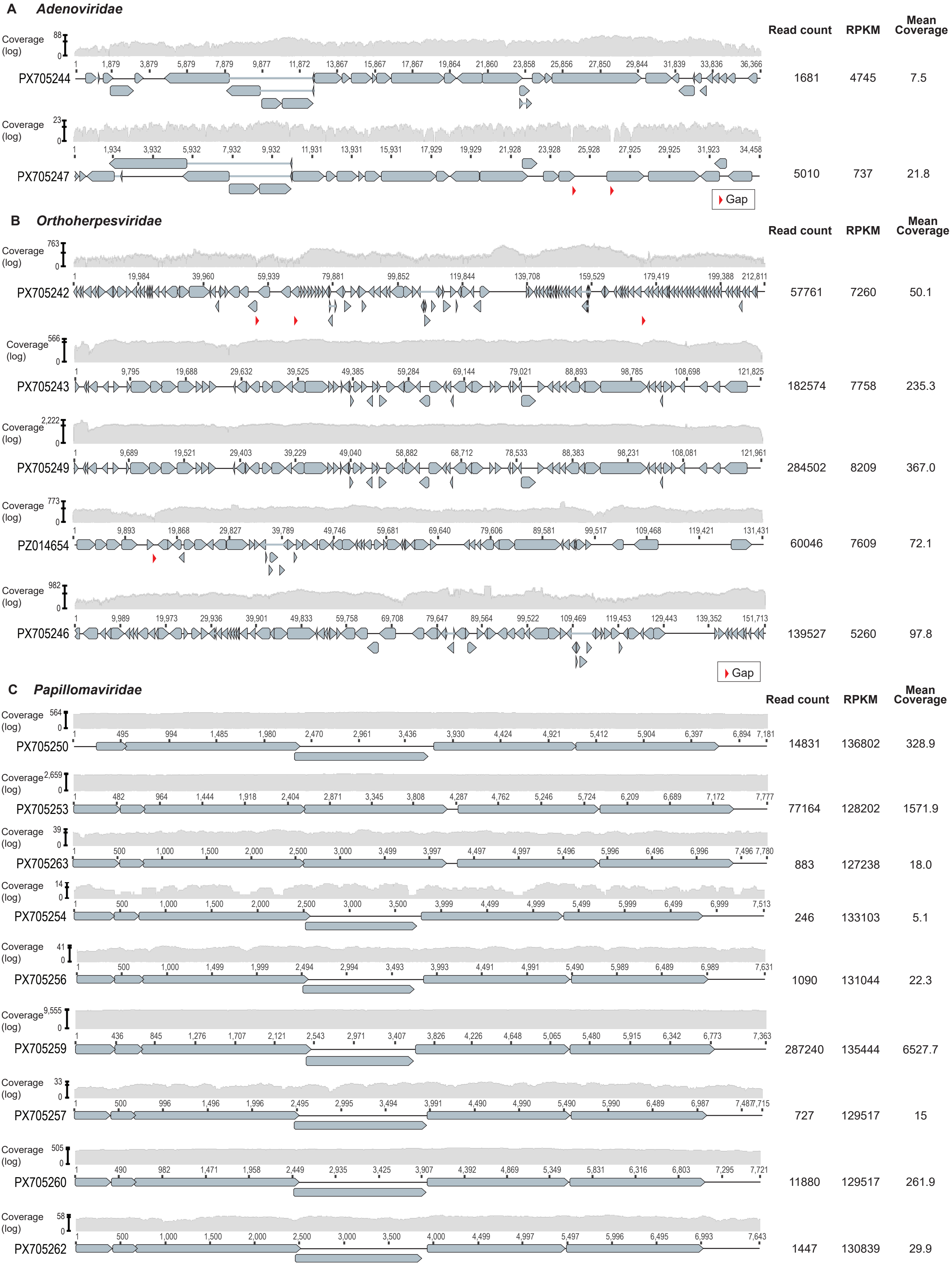
A Adenoviridae
Read count
RPKM
Mean Coverage
Coverage (log)
PX705244
1681
4745
7.5
PX705247
5010
737
21.8
Gap
B Orthoherpesviridae
Read count
RPKM
Mean Coverage
PX705242
57761
7260
50.1
PX705243
182574
7758
235.3
PX705249
284502
8209
367.0
PZ014654
60046
7609
72.1
PX705246
139527
5260
97.8
Gap
C Papillomaviridae
Read count
RPKM
Mean Coverage
PX705250
14831
136802
328.9
PX705253
77164
128202
1571.9
PX705263
883
127238
18.0
PX705254
246
133103
5.1
PX705256
1090
131044
22.3
PX705259
287240
135444
6527.7
PX705257
727
129517
15
PX705260
11880
129517
261.9
PX705262
1447
130839
29.9

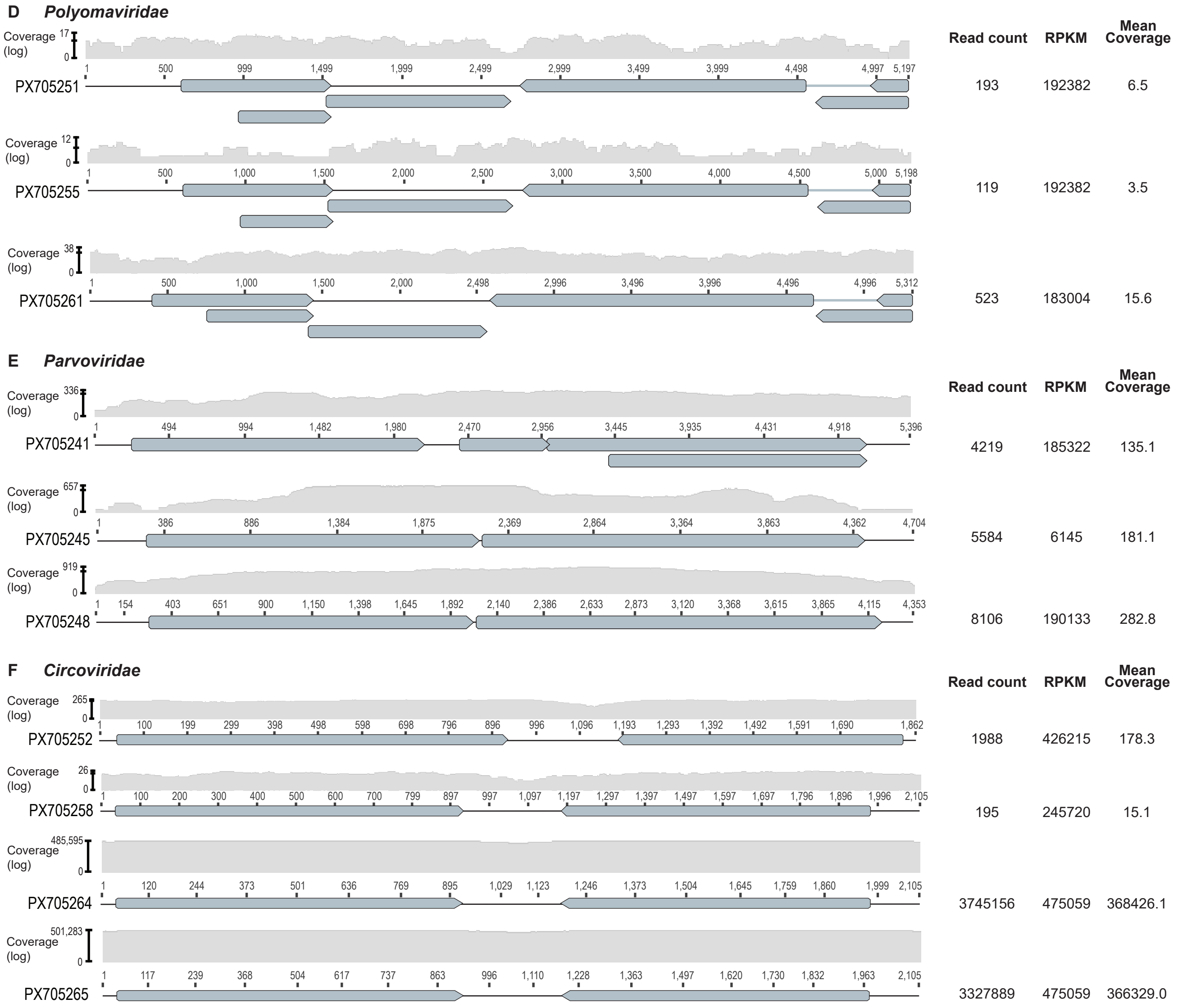

Supplementary Figure 1: Linearized genome organizations, coverage maps, and associated information for all virus sequences characterized in this study. Read count, RPKM (Reads Per Kilobase Million), and mean coverage were computed using raw Illumina sequencing reads from the individual sample in which the sequence was characterized with CoverM.
